# Genetic Variants Related to Increased CKD Progression—A Systematic Review

**DOI:** 10.3390/biology14010068

**Published:** 2025-01-14

**Authors:** Filipe S. Mira, Bárbara Oliveiros, Isabel Marques Carreira, Rui Alves, Ilda Patrícia Ribeiro

**Affiliations:** 1 Department of Nephrology, Unidade Local de Saúde de Coimbra, 3004-561 Coimbra, Portugal; filipemira@netcabo.pt (F.S.M.); ruialves@ulscoimbra.min-saude.pt (R.A.); 2 Faculty of Medicine, University of Coimbra, 3004-504 Coimbra, Portugal; boliveiros@fmed.uc.pt (B.O.); icarreira@fmed.uc.pt (I.M.C.); 3 Laboratory of Biostatistics and Medical Informatics (LBIM), University of Coimbra, 3004-504 Coimbra, Portugal; 4 Center for Innovative Biomedicine and Biotechnology (CIBB), 3004-504 Coimbra, Portugal; 5 Coimbra Institute for Clinical and Biomedical Research (iCBR), 3000-548 Coimbra, Portugal; 6 Center of Investigation on Environment Genetics and Oncobiology (CIMAGO), 3001-301 Coimbra, Portugal; 7 Cytogenetics and Genomics Laboratory, Institute of Cellular and Molecular Biology, 3000-548 Coimbra, Portugal

**Keywords:** chronic kidney disease, end-stage renal disease, single nucleotide polymorphism, estimated glomerular filtration rate (eGFR), genetics, progression

## Abstract

The incidence and prevalence of chronic kidney disease (CKD) are increasing worldwide. CKD is associated with high morbidity, premature mortality, and high healthcare costs. Genetic variants may influence CKD development and progression. This study aimed to identify the associations between allelic variants and CKD progression. We performed a systematic literature review following the Preferred Reporting Items for Systematic Reviews and Meta-Analyses (PRISMA) guidelines. The PubMed, Embase, and Cochrane Central databases were used for data collection. Hereditary causes of CKD were excluded from the analysis. A total of 38 reports were included. The selected studies included cohort studies, case-control studies, and genome-wide association studies (GWASs). The studies involved patients of different ethnicities and with comorbid diseases. Several genetic variants were identified in genes that encode proteins related to metabolic processes, oxidative stress, immune regulation, the renin–angiotensin–aldosterone pathway, and epigenetics, among others. These genetic alterations can affect protein function and lead to renal damage, impacting CKD development and progression. Gene polymorphisms can influence CKD progression. Many of these are population-specific, and their relevance may be influenced by the presence of other diseases and environmental factors. Larger studies are needed to confirm the associations described here.

## Simple Summary

Chronic kidney disease is a progressive disease that results from kidney damage and results in loss of kidney function. Genetic factors, such as allelic variants, can contribute to this disease. This review discusses the link between different allelic variants and CKD progression. Identifying such associations can contribute to the early detection of the disease and support the development of more specific treatment approaches.

## 1. Introduction

Chronic kidney disease (CKD) is a common, multifactorial disease with heterogenous progression to its terminal stage. CKD is defined as the continuous loss of renal function that can be measured by a reduction in the glomerular filtration rate below 60 mL/min/1.73 m^2^ over, at least, a 3-month period. This reduction is the quantification of the irreversible loss of functional nephrons [1]. Although diabetes and hypertension are the main risk factors for CKD, its progression is not the same throughout the affected population [2,3]. Patients with CKD suffer a significant impact on their quality of life due to the inevitable need for food restrictions, the increase in the number of drugs needed to control complications that arise from the disease itself and the eventual need for kidney replacement therapy [4]. As kidney function deteriorates, patients are unable to filter waste products, or to eliminate the amount of urine needed to maintain homeostasis, leading to the accumulation of uremic toxins and ions such as potassium and phosphorus. Besides needing more medication to substitute for the affected basic kidney functions (diuretics, calcium supplements, phosphorus chelating agents, bicarbonate, etc.), when the patient reaches stage-five CKD, renal substitution therapy (in the form of hemodialysis, peritoneal dialysis or renal transplantation) may be essential [4].

It is known that CKD patients suffer from higher genomic instability, which has a direct correlation with increased genetic and chromosomal damage when cells are affected by radiation, as well as less effective DNA repair when damaged [5,6,7]. Therefore, genetic damage may not only be the cause of CKD but also the consequence of the disease itself. Several studies have emphasized the significant genetic impact, not only on the development of end-stage renal disease (ESRD) but also on the progression rate during follow-up in patients with known glomerular disease [8,9].

In order to identify the associations between allelic variants and faster CKD progression, we selected several studies to conduct a systematic review on the topic. Identifying genetic variants that could help stratify a patient’s CKD progression would be useful in understanding the reason for faster CKD progression in certain patients as well as facilitating different treatment strategies with a positive impact on delaying CKD progression related to these genetic variants.

The main outcome measure is the faster CKD progression correlated with the genetic variants identified in the adult population reported in the included studies. CKD progression will be evaluated as the progressive loss of kidney function (decrease in the glomerular filtration rate or increase in serum creatinine) as time passes (months/years). Studies will be registered, and a measure of the effect will be assessed for each study, according to its type. Whenever possible, a common measure of the effect will be computed in order to maximize the number of studies included in the quantitative analysis, even though it may introduce a limitation.

## 2. Materials and Methods

We performed a systematic review that included observational studies (retrospective or prospective cohort studies included), retrospective case-control studies or cross-sectional studies.

### 2.1. Eligibility Criteria

The population studied comprised adult patients with CKD who reported faster progression of their disease. We excluded studies of pediatric patients as well as populations with hereditary and known genetic kidney diseases (autosomal dominant polycystic kidney disease (ADPKD), Alport syndrome, cystinosis, Fabry disease, Gitelman syndrome, nephronophthisis (NPHP)) or glomerular diseases that often have an acute onset and evolution (such as hemolytic uremic syndrome or anti-glomerular basement membrane disease, unless the study described only the chronic follow-up and GFR decay).

Studies were included if they reported genetic variants and compared CKD progression in patients with and without them. Studies must include a control and study population. Studies must not include hereditary or genetically transmitted kidney diseases.

### 2.2. Information Sources and Search Strategy

Data were collected from several databases, specifically PubMed (searched on 18 May 2021), Embase (searched on 8 June 2021) and Cochrane Central (searched on 15 June 2021). Alerts were placed on all the databases and the last update was received on 13 December 2021. The research was repeated on 7 July 2024 and no new results were obtained.

The search strategy used was as follows. Search: ((“Renal Insufficiency, Chronic” [MESH]) OR (Kidney Failure, Chronic [MESH]) OR ((Chronic OR end-stage OR “end stage” OR endstage OR “stage 5”) AND (kidney OR renal OR nephropathy) AND (insufficiency OR disease* OR failure OR disorder OR dysfunction OR impairment)) OR ESRD) AND ((“DNA Copy Number Variations” [MESH]) OR (“Polymorphism, Single Nucleotide” [MESH]) OR (“Polymorphism, Genetic” [MESH]) OR (“Genetic variation” [MESH]) AND ((“copy number” AND (variation OR variant* OR polymorphism* OR change*)) OR CNV) OR (DNA AND ((“copy number” AND (variation OR variant* OR polymorphism* OR change*)) OR CNV)) OR (((“single nucleotide” AND (polymorphism* OR variant OR variation)) OR SNP’s OR SNPs) OR ((genetic OR gene OR genotype) AND (polymorphism* OR variability OR differentiation OR divergenc* OR diversit* OR variance OR loci)))). Filters: Clinical Study, Evaluation Study, Observational Study, Humans, English, French, Portuguese, Spanish, Adult: 19+ years, Young Adult: 19–24 years, Adult: 19–44 years, Middle Aged + Aged: 45+ years, Middle Aged: 45–64 years, Aged: 65+ years, 80 and over: 80+ years.

### 2.3. Study Screening

The screening process involved four of the authors, who are identified by their initials. It began after the exclusion of duplicate studies through an Excel tool designed for the effect (B.O.), which performs text comparisons across spreadsheets containing search results from different sources. The results were normalized with an R application built for that purpose. The final step in this procedure generated an Excel spreadsheet, which was independently assessed by two reviewers (F.M. and I.R.), who decided on inclusion based on the title and abstract. If the decision was made to exclude a study, then the reason was specified.

The individual spreadsheets from each reviewer were automatically compared in a new Excel workbook to assess the inter-rater concordance through Cohen’s kappa (B.O.) and to identify eventual opinion divergences between reviewers. If an agreement was not reached, then a third reviewer (I.C.) was involved to make the final decision. This procedure was repeated after a full-text reading by both reviewers (F.M. and I.R.), which would depend on a third reviewer (I.C.) as well if an agreement was not reached. Cohen’s kappa was also calculated in this step (B.O.) to assess the inter-rater agreement.

The data extracted included the study identification (1st author, year followed by sequential letter beginning with “a” if more than one study from the same author/year), study type, sample size, CKD progression (GFR decrease/unit), cause of CKD, follow-up time, outcomes, genetic variants, minor allele frequency, study exclusion criteria, and country where it was performed.

A qualitative data synthesis was performed considering the studies included after the screening. This procedure was performed to establish the array of genetic variants identified in order to have a prognostic value for ESRD, reporting separately the studies with a clear and positive identification, a negative one, or an inconclusive response.

This study was designed according to the Preferred Reporting Items for Systematic Reviews and Meta-Analyses (PRISMA) statement and its registration in the PROSPERO database of the National Institute for Health and Care Research was performed at inception (CRD42022299144) [10].

A qualitative descriptive approach was used to summarize the study characteristics and outcomes. There was a plan to perform a quantitative synthesis and subgroup analyses, but since the outcomes were heterogenous, it was not performed in the end.

### 2.4. Risk of Bias

The risk of bias was assessed independently by two authors (F.M. and I.R.) using the Newcastle–Ottawa Scale for observational studies [11], which has a scoring scale of 0 to 9 stars. Articles were classified as “High Quality” (8 to 9 stars), “Medium Quality” (4 to 7 stars) or “Low Quality” (less than 4 stars). 

## 3. Results

### 3.1. Identification of Relevant Studies

**Figure 1:**
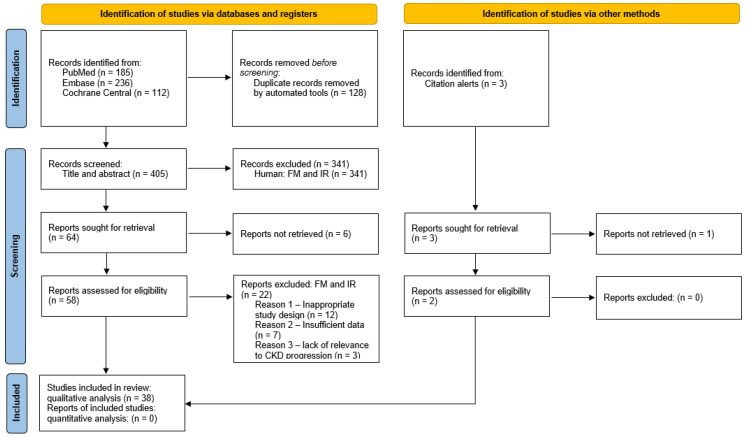
PRISMA flowchart for the systematic review of the literature.

A comprehensive search strategy initially retrieved 533 articles, which, after removing duplicates, resulted in 405 unique studies. Following a meticulous screening process based on predefined inclusion and exclusion criteria, 38 studies were deemed eligible for inclusion in this systematic review (Figure 1, Table 1, Supplementary Table S1) [12,13,14,15,16,17,18,19,20,21,22,23,24,25,26,27,28,29,30,31,32,33,34,35,36,37,38,39,40,41,42,43,44,45,46,47,48,49]. The reasons for exclusion encompassed an inappropriate study design, insufficient data, lack of relevance to CKD progression, and the presence of topics irrelevant to our study.

### 3.2. Characteristics of Included Studies and Quality

The selected studies comprised a diverse array of research designs, including cohort studies (18), case-control studies (15), and genome-wide association studies (GWASs) (5), exploring genetic determinants associated with the progression of chronic kidney disease (CKD). There was a total of 26,967 patients with CKD and more than 150 genetic variants studied.

None of the studies identified as relevant were meta-analyses. These investigations spanned various populations and ethnicities, providing insights into the genetic underpinnings influencing CKD progression across different demographic groups.

Analysis of the included studies revealed a spectrum of genetic variants potentially linked to the progression of CKD.

Of the 38 studies, only 14 measured the outcome/exposure objectively (CKD progression) and were considered to be of high quality. In terms of comparability, only eight used CKD progression to assess comparability. The remaining studies were of medium quality, although the participant selection process was generally robust in most studies (Supplementary Table S2).

### 3.3. MYH9 and APOL1 Polymorphisms

Polymorphisms in the non-muscle heavy chain 9 (*MYH9*) and apolipoprotein L1 (*APOL1*) genes are known to be associated with the risk of CKD development, particularly in African-descendent populations [39]. However, the two genes co-segregate in many populations, making it difficult to differentiate between the two association signals [39]. Colares et al. evaluated the effect of *MYH9* and *APOL1* gene polymorphisms on the risk of CKD in a cohort of lupus nephritis patients [39]. The cohort included 196 Brazilian patients, corresponding to a highly mixed population, including African, American Indian, and European ancestry. Among all the polymorphisms analyzed, only *MYH9* rs3752462 was associated with the primary outcome, defined as the doubling of serum creatinine or the need for renal replacement therapy (adjusted hazard ratio [HR] 3.72, 95% confidence interval [CI] 1.47–9.38, *p* = 0.005). The presence of at least one rs3752462 risk allele was significantly associated with the risk of CKD, even after adjusting for the estimated creatinine clearance and African autosomal ancestry (HR 2.0, 95% CI 1.2–3.4, *p* = 0.01). The results were maintained after adjusting for the different ancestry covariates, indicating that in this cohort, *MYH9*, but not *APOL1*, gene polymorphisms confer an increased risk of CKD in lupus nephritis patients, independently of race [39]. *MYH9* rs3752462 was also identified as an independent risk factor for CKD in a cohort of 592 Spanish Caucasian individuals aged 55 to 85 years [45]. Carriers of the T allele (TC + TT genotypes) had a lower mean eGFR compared with the CC homozygotes (77 ± 18 vs. 83 ± 19, *p* = 0.02) [45].

Another *MYH9* gene polymorphism, rs4821480, was evaluated in a retrospective cohort of 154 non-diabetic ESRD patients and 123 age-matched healthy controls from Saudi Arabia [16]. rs4821480 showed a significant association with the risk of ESRD development, including after adjusting the model for the age and sex variables (OR 3.867, 95% CI 1.38–10.82, *p* = 0.01 and OR 3.85, 95% CI 1.37–10.73, *p* = 0.01, respectively) [16].

In a genome-wide association study (GWAS) from 2019, several variants of the *APOL1* locus were associated with ESRD but not with CKD, including the *APOL1* rs73885319 and rs60910145 [49]. The study included multi-ethnic CKD patients (4150 mild-to-moderate CKD and 1105 ESRD) and non-CKD controls, totaling 41,041 participants with kidney phenotype information. Knowing that not all individuals carrying high-risk *APOL1* variants experience CKD progression, Chen and colleagues analyzed the potential modifying risk factors that may interfere with the *APOL1* high-risk genotype and CKD progression [34]. The study included 693 individuals who participated in the African American Study of Kidney Disease and Hypertension (AASK) trial, representing an African American population with hypertension-related CKD. The results confirmed the previously reported findings that individuals with the *APOL1* high-risk genotype had an increased risk of CKD progression. The risk was 1.88-fold higher than in the low-risk group after adjusting for potential confounding variables (95% CI 1.46–2.41, *p* = 0.001) [34]. However, among the 21 clinical and laboratory parameters studied, the authors failed to identify patient characteristics that modify the association between *APOL1* gene polymorphisms and CKD progression. Nevertheless, obesity and increased urinary secretion of urea nitrogen were associated with a lower risk of *APOL1*-mediated CKD progression but were not robust enough to pass the sensitivity analysis [34].

### 3.4. Genes Involved in Oxidative Stress

The SNPs in superoxide dismutase 1 and 2 (*SOD1* and *SOD2*) were evaluated in two studies [28,48]. The first study enrolled 671 ESRD patients and 780 controls without CKD of Han Chinese origin [28]. DM patients carrying the *SOD2* exon 2 CC genotype had a significantly higher risk of developing ESRD (OR 0.699, 95% CI 0.52–0.94, *p* = 0.018). Interestingly, in patients without DM, the TT genotype was related to a lower risk of developing ESRD (OR 0.69, 95% CI 0.51–0.93, *p* = 0.014) [28]. In another study in the Spanish population, the *SOD1* and *SOD2* polymorphisms failed to show an association with CKD [48]. Nevertheless, *SOD1* rs17880135, rs202446, and rs1041740 showed an association with the albumin and phosphorus levels, while *SOD2* rs4880 showed an association with the resistance index erythropoietin, C-reactive protein, and ferritin, which are biochemical parameters characteristic of CKD [48].

The contribution of proliferator-activated receptor (PPAR) γ (*PPARG*) polymorphisms was accessed in two studies [23,28]. The first studied two variants, rs1801282 and rs3856806, and found that the rs1801282 CC genotype occurred more frequently in the controls than in the CKD patients (*p* = 0.028). Accordingly, the rs1801282 and rs3856806 CC genotypes were protective against ESRD (OR 0.78, 95% CI 0.61–0.99, *p* = 0.04), while CKD patients with the rs1801282 TT genotype and rs3856806 GG genotype showed a higher risk of ESRD (*p* < 0.001) [28]. In another study with a prospective cohort design of patients with T2D from Thailand, Pro12Ala (codon 12, exon 2) variants of *PPARG2* were not related to the ESRD incidence [23]. In a subgroup analysis of patients with baseline GFR > 60 mL/min/1.73 m^2^, patients with the Pro12Ala genotype had a significantly higher CKD incidence (*p* = 0.034), even after adjusting for established CKD risk factors (HR 3.75, 95% CI 1.10–12.81, *p* = 0.047) [23].

A Malaysian retrospective case-control study involving 600 T2D patients, of whom 300 were CKD patients and 300 were controls, found that the *PPARGC1A* rs8192678 polymorphism, the gene that encodes a transcriptional coactivator of PPAR-γ, was significantly associated with CKD [15]. The authors found a synergistic interaction of the *PPARGC1A* rs8192678 TT genotype and smoking, leading to a 30-fold increased risk of CKD (versus 3-fold and 20-fold for *PPARGC1A* rs8192678 and smoking’s individual risk factors, respectively) [15]. Additionally, several gene–environmental interactions were established, and *PPARGC1A* rs8192678 was significantly associated with sex (male gender), smoking, high-density lipoprotein (HDL), and waist circumference, representing a higher CKD probability for participants with high-risk factors [15].

The role of the glutathione peroxidase (*GPX*) gene in CKD was evaluated in three studies [28,33,48]. Chao et al. did not detect a significant effect of *GPX1* rs1050450 on the risk of developing ESRD. However, subgroup analysis showed that patients with simultaneous *GPX1* rs1050450 CC genotype and *PPARG* rs1801282 GG genotype had a significantly lower ESRD risk (*p* < 0.001) [28]. In a Spanish cohort, *GPX1* rs17080528 was significantly associated with CKD susceptibility (OR = 1.87, *p* = 0.001), and *GPX4* rs713041 was associated with the hypertension incidence among CKD patients [48]. In the same study, rs17080528 from *GPX1* showed an association with creatinine, the glomerular filtration rate, and phosphorus biochemical parameters, while rs713041 from *GPX4* was associated with phosphorus and ferritin [48]. In another study, which included 1385 T1D patients from three different cohorts, the T allele of *GPX1* rs3448 was associated with the incidence of renal events (HR 1.81, 95% CI 1.16–2.84, *p* = 0.008) and ESRD (HR 3.34, 95%CI, 1.69–6.98, *p* = 0.0004) [33]. The model was adjusted for different parameters, such as age, sex, diabetes duration, HbA1c, blood pressure, use of ACE inhibitors, and diabetic retinopathy stages. The same allele was also associated with higher plasma isoprostane and advanced oxidation protein product (AOPP) concentrations [33].

Variants in the *CYBA* gene, encoding the regulatory subunit p22^phox^ of NADPH oxidase, were studied in three independent prospective cohorts of T1D Caucasian patients (SURGENE, GENEDIAB, and GENESIS studies) [38]. The major G allele of rs9932581 was significantly associated with the incidence of renal events, defined as new cases of microalbuminuria or the progression to a more severe stage of nephropathy (HR 1.59, 95% CI 1.17–2.18, *p* = 0.003) in T1D patients from SURGENE. The same allele was associated with established/advanced nephropathy (OR 1.52, 95% CI 1.22–1.92, *p* = 0.0001) and with the progression to ESRD (HR 2.01, 95% CI 1.30–3.24, *p* =0.001) in the GENEDIAB and GENESIS cohorts. Additionally, the G allele was associated with higher plasma AOPP and myeloperoxidase (MPO) concentrations, lower eGFR, and arterial hypertension [38].

Hattori and colleagues conducted two studies, one longitudinal and the other cross-sectional, to evaluate the association between metallothionein 2A (*MT2A*) rs28366003 and the risk of CKD in a cohort of 2774 DM patients residents in Nagoya City, Japan [31]. *MT2A* rs28366003 showed a significant association with CKD, before and after adjusting for general and possible confounding parameters [31].

### 3.5. Metabolism-Related Genes

In a multicenter, observational, prospective study by Valls et al., the authors analyzed 79 SNPs of proteins involved in mineral metabolism in a cohort of 2445 CKD cases and 559 controls [24]. After removing from the analysis the SNPs missing in the controls and adjusting for confounding variables, 12 SNPs showed a significant association with CKD (Table 1, Supplementary Table S1). Furthermore, the authors identified eight SNPs that were statistically significant for CKD risk prediction, even when classical risk factors were considered. Using another model that took into consideration the interactions between SNPs, hypertension, and diabetes, the authors identified a hypothetical high-risk patient profile (male, non-Caucasian, with diabetes and hypertension), where the presence of the five SNPs (*SPP1* s1126616, *MMP3* rs35068180, *BGLAP* rs1800247, *MGP* rs4236, and *CYP24A1* rs2248359) increased the risk of CKD by six times [24].

The effect of the nitric oxide synthase 3 (*NOS3*) gene was evaluated in a retrospective case-control study [15]. The authors showed that *NOS3* rs2070744 was associated with CKD development [15]. Furthermore, *NOS3* rs2070744 showed a significant interaction with smoking, waist circumference, and HDL, meaning that the effect of the genotype differences was more potent in the high-risk groups [15]. In the same study, the authors also assessed the role of the potassium voltage-gated channel subfamily Q member 1 (*KCNQ1*) SNPs rs2237895 and rs2283228. Both SNPs were associated with CKD development, and the *KCNQ1* rs2283228 CC genotype was significantly associated with CKD progression (adjusted RR 1.65, 95% CI 1.11–12.45, *p* = 0.013) [15]. *KCNQ1* rs2283228 had a synergistic effect with sex and BMI, having a greater impact on men and obese patients [15].

Fatumo and colleagues developed a GWAS for the eGFR in a cohort of 3288 East Africans and a validation cohort of 8224 African Americans [12]. Two loci were significantly associated with the eGFR, corresponding to glycine amidinotransferase (*GATM*) rs2433603 and hemoglobin subunit beta (*HBB*) rs141845179. *GATM* rs2433603 was replicated in the second cohort (*p* = 0.00073), while the HBB locus was considered rare or monomorphic in other populations. Nevertheless, another SNP, rs334, a close proxy for rs141845179 at the *HBB* locus and representing the same eGFR signal, allowed the replication of the *HBB* loci in the second cohort (*p* = 0.017) [12].

Cytochrome P450 (CYP450) proteins metabolize many reactions involved in drug metabolism [13]. In a multicenter, observational, prospective cohort study of 124 CKD patients in Malaysia, the *CYP3A5* rs776746 genetic polymorphism, encoding one of the most common CYP450 proteins among the Asian population, was associated with rapid CKD progression (adjusted OR 4.190, 95% CI 1.268–13.852). Rapid CKD progression corresponded to a sustained decline in the eGFR of more than 5 mL/min/1.73 m^2^/year. While the baseline eGFR did not differ significantly between the *CYP3A5* rs776746 genotypes, at the end of the follow-up, the eGFR was significantly lower in patients with the CYP3A5*3/*3 genotype compared with patients carrying the CYP3A5*1/*1 genotype and CYP3A5*1/*3 genotype (45.7 ± 20.9 mL/min/1.73 m^2^, 58.2 ± 32.6 mL/min/1.73 m^2^, and 63.3 ± 34.7 mL/min/1.73 m^2^, respectively, *p* = 0.03) [13].

Mori et al. evaluated five *HSD11B1* SNPs (rs11799643, rs17389016, rs4844880, rs846910, rs846906) in 466 T1D Brazilian patients [14]. After adjusting for confounding variables, the minor C allele of rs17389016 was associated with diabetic kidney disease (DKD) (OR 1.90, 95% CI 1.07–3.37, *p* = 0.028), while the minor T allele of rs11799643 was associated with diabetic retinopathy (DR) (OR 0.52, 95% CI 0.28–0.96, *p* = 0.036). The minor T allele of rs846906 was associated with a higher prevalence of arterial hypertension, body mass index (BMI), and waist circumference, conferring an increased risk of a lower estimated glucose disposal rate (eGDR) (OR 1.23, 95% CI 1.06–1.42, *p* = 0.004) [14].

ATP-binding cassette subfamily G member 8 (*ABCG8*) is an important gene in liver metabolism [37]. The association between variant T400K (rs4148217, C > A, exon 8) and severe kidney outcomes in T2D patients was evaluated in a French retrospective cohort study [37]. T400K was significantly associated with a higher risk of renal events, defined as a doubling of the serum creatinine concentration or ESRD (HR 1.52, 95% CI 1.05–2.21, *p* = 0.03) in the fully adjusted model. The results were validated in a second cohort that showed an association between T400K and a higher prevalence of ESRD (OR 1.63, 95% CI 1.05–2.54, *p* = 0.03) [37].

Regarding metabolic-related genes, the rs641738 variant in the locus that contains the membrane-bound O-acyltransferase domain-containing 7 (*MBOAT7*) and transmembrane channel-like 4 (*TMC4*) genes were also associated with the CKD risk [19].

### 3.6. Renin–Angiotensin–Aldosterone System Genes

Two studies addressed the role of renin–angiotensin–aldosterone system (RAAS)-related genes in CKD and diabetic nephropathy [36,44]. In a retrospective cohort study from 2015, 523 Caucasian and 1490 African-descendant patients with different stages of CKD were enrolled [36]. Among 12 candidate genes, the *ACE* gene was significantly associated with eGFR decline in Caucasians, while *ACE2*, *AGTR2* and the RAAS pathway were significantly associated with eGFR decline in African-descendants [36]. Gene and pathway-based analyses of renal events showed that *AGT*, *RENBP*, and the entire RAAS pathway were significantly associated with renal events among both ancestries. In this study, the authors did not find any SNP associated with CKD progression [36].

### 3.7. Immune-Related Genes

Mannose-binding lectin 2 (*MBL2*) was evaluated in two Chinese studies [17,22]. The first enrolled a total of 1007 immunoglobulin A nephropathy (IgAN) patients and 121 healthy controls divided between a discovery and a validation cohort [22]. The rs1800450 in *MBL2* was associated with an increased risk of ESRD in the fully adjusted model (HR 9.64, 95% CI 2.40–38.71, *p* = 0.001) and the validation cohort (HR 15.91, 95% CI 3.27–77.34, *p* = 0.001). In the discovery cohort, rs1800450 increased the risk of ESRD by 2.21-fold. Patients with rs1800450 lacked expression of the MBL protein in both the serum and renal tissue, suggesting an inactivation of the MBL pathway. Using a previously published risk score that includes clinical parameters such as age, eGFR, and two pathology scores, the rs1800450 AA genotype had a combined effect with such parameters, resulting in an increased 10-year risk of ESRD by 37-fold [22]. The other study included 77 T2D patients and found that patients with the rs1800450 GG genotype had a higher serum MBL level compared to those with the GA genotype [17]. In a second independent cohort of 133 T2D patients, the rs1800450 GA genotype was an independent protective factor for ESRD (HR 0.485, 95% CI 0.237–0.991, *p* = 0.047), adjusted for different clinical parameters [17].

Complement C3 (*C3*) plays an important role in the immune system and is also involved in kidney diseases [18]. The association of *C3* rs2230199 with CKD was investigated in two retrospective cohorts, one composed of 514 CKD patients (stages 3–5) and 454 matched controls, and the second of 269 glomerulonephritis (GN) patients [18]. Although the minor allele frequency was significantly higher in the CKD patients compared with the control group (*p* = 0.008), there was no significant association between rs2230199 and CKD progression or mortality. However, subgroup analysis showed a significant association between rs2230199 and CKD progression in the IgAN group for both heterozygous and homozygous variants of the minor allele (HR 1.9, 95% CI 1.1–3.1, *p* = 0.018 and HR 2.8, 95% CI 1.2–6.2, *p* = 0.014, respectively), with major allele homozygosity showing a protective effect (HR 0.41, 95% CI 0.24–0.68, *p* = 0.001) [18].

The *BTN2A1* gene, which belongs to the BTN superfamily, is another immune-related gene studied in a population-based cohort of CKD patients from Japan [43]. The minor T allele of rs6929846 was significantly associated with an increased risk of CKD in the multivariate logistic regression (OR 2.00, 95% CI 1.38–2.91, *p* = 0.0003). The rs6929846 T genotypes (CT and TT) were also associated with a higher serum concentration of creatinine and a lower eGFR [43].

A four-SNP genetic risk score associated with IgAN progression that included immune-related genes was identified by Shi et al. [25]. The discovery cohort included 613 Chinese patients diagnosed with IgAN and involved the genotyping of 20 candidate SNPs selected from previous GWASs. The model contained the variants *ITGAM-ITGAX* s11150612, *ST6GAL* rs7634389, *HORMAD2* rs2412971, and *HLA-DQ/DR* rs2856717. The genetic risk score was associated with disease progression in the adjusted clinical risk model (HR 1.29, 95% CI 1.03–1.62, *p* = 0.03) and clinical-pathologic risk model (HR 1.35, 95% CI 1.03–1.77, *p* = 0.03). Patient stratification according to the identified risk score showed that patients in the middle-risk group had a 2.12-fold increase in the risk of disease progression (HR 2.12, 95% CI 1.33–3.40, *p* = 0.002), whereas patients in the high-risk group had a 3.61-fold risk increase (HR 3.61, 95% CI 2.00–6.52, *p* < 0.001) when compared to patients in the low-risk score [25]. The authors stated that the underlying pathogenic mechanism of this score may be the autoantigen formation and antigen presentation that impacts IgAN development and progression [25]. The polymorphisms of HLA class I and II antigens were previously investigated in a Taiwanese cohort of ESRD patients; however, results did not reach significance in the adjusted models [35].

### 3.8. Epigenetics

The rs653747 located in the RNA-coding gene *LINC00923* was associated with eGFR decline among the non-diabetic population in a GWAS [26]. The study identified three additional SNPs associated with the rate of renal function decline, but only *LINC00923* rs653747 was replicated in a second cohort. *LINC00923* rs653747 showed significance among Black and White non-diabetic CKD patients and was also associated with the risk of incident ESRD [26].

*EP300*, a transcriptional coactivator, and *SIRT1*, an epigenetic regulator, were associated with DKD in a Chinese cohort of T2D patients with or without CKD [27]. The G allele of *EP300* rs20551 was correlated with an increased risk of DKD development in T2D patients. Subgroup analysis showed that this genotype conferred an increased risk among female patients and patients less than 65 years of age. Additionally, T2D patients with both *EP300* rs20551 G alleles and the *SIRT1* rs4746720 TT genotype had a higher risk of developing serious DKD [27].

Ozdemir et al. studied the effect of germline *MTHFR* C677T mutations in CKD in a cohort of 228 CRF patients and 212 healthy individuals [41]. *MTHFR* C677T mutations led to a 2.53-fold increase in the risk of CKD (OR 2.513, CI 1.78–3.55, *p* < 0.0001). This association was attributed to the reduced enzymatic activity triggered by the C677T mutation, which results in a global DNA hypomethylation [41].

### 3.9. Other Genetic Variations

Two studies addressed the role of the transmembrane channel *Aquaporin 11* (*AQP11*) rs2276415 in CKD [20,30]. Choma et al. evaluated the role of *AQP11* rs2276415 in a retrospective case-control study of 1075 diabetic patients and 1619 controls [30]. *AQP11* rs2276415 was significantly associated with the prevalence of acute kidney injury (AKI) and CKD only in the diabetic patients (*p* = 0.036). After adjusting for age, sex, baseline serum creatinine, and comorbid diseases, the multivariate logistic regression showed that T2D patients with the *AQP11* rs2276415 polymorphism were at a 2.25 times higher risk of developing CKD compared with T2D patients with the wild-type allele (*p* = 0.006). This increased risk was absent in the non-diabetic patients [30]. The results were validated in an independent cohort. The combined data from both studies confirmed that *AQP11* rs2276415 confers an increased CKD risk in diabetic patients (OD 1.81, *p* < 0.001) but not in the non-diabetic population (*p* = 0.198) [30]. Furthermore, CKD patients who are more than 55 years of age and who carry the *AQP11* minor allele have a significantly higher rate of eGFR decline compared with wild-type allele carriers (*p* = 0.023) [30]. The *AQP11* rs2276415 variant corresponds to the substitution of a glycine at position 102 by a serine in the functional, pore-forming region of AQP11 [30]. Three-dimensional modeling revealed the possibility of structural alterations in the AQP11 pore-forming region that can result in AQP11 dysfunction and a higher risk of developing CKD in diabetic patients carrying the risk allele [30].

In a prospective cohort study of 620 Chinese CKD patients, the *AQP11* rs2276415 variant was significantly associated with an increased risk of CKD progression in a univariate (HR 3.20, 95% CI 2.35–4.36, *p* < 0.001) and multiple Cox regression model adjusted for the risk factors (HR 1.92, 95% CI 1.31–2.84, *p* = 0.001) [20]. In this study, the association of *AQP11* rs2276415 with CKD progression was verified in both diabetic and non-diabetic patients. *AQP11* rs2276415 increased the risk of ESRD by 3.19 (95% CI 1.47–6.94, *p* = 0.003) [20].

Thirty-six genetic variants associated with T2D, obesity, and fasting plasma glucose were evaluated in a large cohort of 2755 T2D patients from China and validated in two independent cross-sectional cohorts [32]. Three SNPs were identified (*G6PC2* rs478333 and *CDKAL1* rs7754840 and rs7756992) and used to calculate a risk score. Individuals with increasing risk scores had a higher risk of CKD development (HR 1.17, 95% CI 1.1–1.26, *p* = 6.3 × 10^−6^). In the logistic regression, rs478333 was associated with a rapid decline in the eGFR (OR 1.16, 95% CI 1.02–1.34, *p* = 0.029). The results for two of the three SNPs were validated in the other two independent cohorts (rs478333 and rs7754840) [32].

The fat mass and obesity-associated gene (*FTO*) rs17817449 variant was evaluated in a case-control study from the Czech Republic that involved patients with CKD, patients with ESRD, and population-based controls [46]. There was a significant association between the G genotypes of rs17817449 and the risk of CKD and ESRD (GG versus TT genotype: OR 1.37, 95% CI 1.20–1.56, *p* < 0.0001). rs17817449 was also associated with an early onset of hemodialysis and kidney transplantation in 3.3 and 2.5 years, respectively [46].

Kidney-structure-related genes were also evaluated in a study that involved T2D African-descendent patients [29]. Three SNPs were identified as significant for the risk of developing T2D-related ESRD and validated in replication cohorts and a study meta-analysis. Two of the identified SNPs correspond to intronic variants (rs116139597, OR 1.28, 95% CI 1.13–1.46, *p* = 1.2 × 10^−4^, and rs115912771, OR 0.63, 95% CI 0.49–0.80, *p* = 1.6 × 10^−4^), located in the *CD2AP* region, which overlaps with a small portion of the adjacent gene *ADGRF2*. The third identified SNP, rs7185763, is located upstream from the *MMP2* gene (OR 0.87, 9% CI 0.81–0.95, *p* = 9.5 × 10^−4^) [29]. A second analysis was performed by removing *APOL1* renal-risk genotype carriers, and five additional variations were identified: *CLDN8* rs55884670, *COL4A3* rs34505188, *NPHP3-ACAD11* rs78174962, *AC009495.3* rs6742727, and *ARHGAP24* rs10433935 [29]. Bioinformatic analysis showed that rs10433935 was strongly associated with the expression level of ARHGAP24 in artery-tibial tissue and that rs6742727 and rs7185763 were associated with the transcript abundance of the *GALNT3*, *AC00949.2*, and *IRX6* genes. Finally, using the prediction scores, *COL4A3* rs34505188, *WNK4* rs57737815, and *ARHGAP24* rs10433935 were predicted to be potential pathogenic variants [29].

Two studies also reported a significant association between Matrix Gla protein (*MGP*) gene polymorphisms and CKD in Spanish and Turkish cohorts [47,48].

### 3.10. GWAS in the Diabetic Population

In the diabetic population, the risk of diabetic kidney disease and ESRD is influenced by genetic variations and gender [40,42]. To identify the sex-specific genetic risk factors that influence ESRD development in T1D patients, Sandholm and colleagues conducted a GWAS including 3652 Finnish patients [40]. In women, two highly correlated SNPs on chromosome 2q31.1, rs4972593 and rs530673, reached genome-wide significance for ESRD (OR 2.39, 95% CI 1.75–3.25, *p* = 3.023, and OR 2.38, 95% CI 1.75–3.23, *p* = 3.523, respectively) and remained significative after adjusting for covariates. The association between rs4972593 in women was validated in three additional T1D cohorts. Interestingly, no loci reached genome-wide significance in men. Furthermore, the identified SNPs were not associated with ESRD in women with T2D. rs4972593 is located between the genes that code for the Sp3 and CDCA7 transcription factors. In silico analysis revealed the loss of several transcription-factor-binding sites through the presence of the minor allele A, including binding sites for E-box and hypoxia-inducible factors. The authors also predicted eight estrogen-responsive elements within 5k base pairs up- and downstream of rs4972593. Finally, SP3 was found to be differently expressed in the glomeruli in a sex-specific manner [40]. Overall, this study identified a sex-specific susceptibility locus for the ESRD risk in the T1D female population.

Later, Sambo et al. used the same cohort of patients (FinnDiane study) to explore the genetic variants associated with diabetic nephropathy and ESRD in T1D patients, regardless of gender [42]. Five SNPs (rs12137135 between *WNT4* and *ZBTB40*, rs17709344 between *RGMA* and *MCTP2*, *MAPRE1P2* rs1670754, rs12917114 between *SEMA6D* and *SLC24A5*, and *SIK1* rs2838302) were significantly associated with the ESRD risk independently of potential confounding factors. rs17709344 between *RGMA* and *MCTP2* was validated in the independent cohorts and in a meta-analysis of those cohorts (*p* = 0.012) [42].

## 4. Discussion

CKD is a progressive disease that ultimately leads to kidney failure, often requiring renal replacement therapy [50]. CKD-related mortality is increasing, and CKD is currently classified as the third fastest-growing cause of death globally [51]. Projections suggest that by 2024, CKD will rank as the fifth leading cause of years of life lost globally [52].

Clinical biomarkers such as the eGFR and albuminuria are well established for CKD diagnosis but have limited power in predicting CKD progression [53]. CKD is a heterogeneous and multifactorial disease with progression influenced by several factors, such as age, ethnicity, baseline eGFR, and proteinuria [54]. Genetic factors also play a significant role, contributing up to 30% of CKD cases [8]. GWASs have identified several genetic variants associated with CKD development and progression [55]. Integrating these genetic variants into diagnostic strategies could enhance disease management and enable personalized treatments [56].

**Figure 2:**
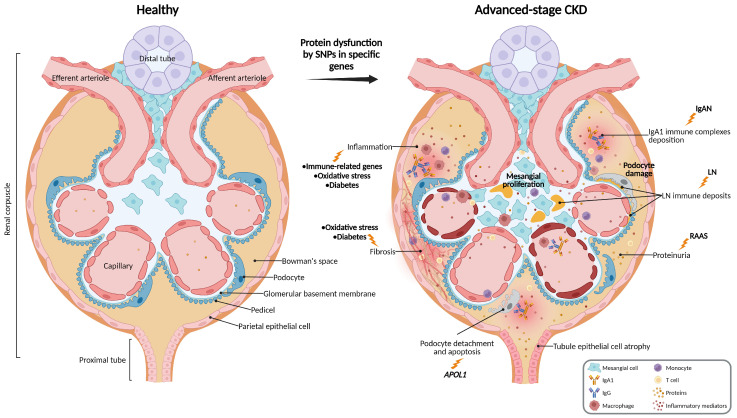
Glomeruli changes in advanced-stage chronic kidney disease. Allele variations in specific genes can cause protein dysfunction, promoting molecular and cellular alterations that ultimately lead to renal tissue damage and CKD progression. Variations in immune-related and oxidative stress genes promote local inflammation by releasing pro-inflammatory mediators and promoting tissue infiltration by macrophages, monocytes, and T cells. Local inflammation promotes tubular atrophy and interstitial fibrosis. Alterations that affect elements of the renin–angiotensin–aldosterone system (RAAS) promote glomerular hypertension, podocyte hypertrophy, and proteinuria. Other specific gene alterations, such as APOL1 mutations, promote podocyte damage and loss. Allele alterations can also promote chronic kidney disease progression in the context of other underlying diseases. In lupus nephritis (LN), immune complex deposition causes inflammation, fibrosis, direct podocyte damage, and endothelial injury. In IgA nephropathy (IgAN), there is the formation and deposition of immune complexes that induce the proliferation of mesangial cells, attack immune cells, and podocyte damage and apoptosis. In the context of diabetic nephropathy, such genetic alterations can accelerate renal dysfunction, with increased inflammation, oxidative stress, and fibrosis. The different mechanisms ultimately lead to renal dysfunction and a vicious cycle, promoting chronic kidney disease progression. IgAN, IgA nephropathy; LN, lupus nephritis; RAAS, renin–angiotensin–aldosterone system. Created with Biorender.com.

Some of the genetic variants associated with CKD are population-specific. Gene variants in *APOL1*, frequent among African-descendent populations, confer resistance to *Trypanosoma brucei* [57]. However, such mutations affect endosomal trafficking and autophagic flux, promoting podocyte and nephron loss, glomerulosclerosis, and ultimately, CKD progression (Figure 2) [58]. This discovery explained the higher rates of ESRD among patients with African ancestry. Initial studies described genetic variants of the *MYH9* gene [59,60], but subsequent studies revealed a high linkage disequilibrium of *MYH9* with *APOL1*, with the latter having a higher association with kidney disease [57,61]. Accordingly, the association of *MYH9* with CKD disappeared after adjusting for the *APOL1* variants [57]. Nevertheless, *MYH9* mutations can cause a monogenic disease with kidney involvement, and *MYH9* rs3752462 was considered an independent risk factor for CKD, nephrotic syndrome, and ESRD in case-control studies from different populations [39,45,62].

Two *APOL1* haplotypes were identified, G1 and G2. G1 corresponds to two missense variants in nearly absolute linkage disequilibrium, rs73885319 (S342G) and rs60910145 (I384M), and G2 correspond to an in-frame deletion del.N388/Y389 (rs71785313) [57]. The two alleles G1 and G2 are mutually exclusive [57]. However, only a subset of individuals with *APOL1* risk variants develop kidney disease, highlighting how other contributing factors trigger CKD progression or protect individuals from the disease [63]. Comorbid infections and inflammatory diseases have been proposed as a potential second hit in these patients [63,64]. Lupus nephritis is a common and severe complication of systemic lupus erythematosus characterized by immune complex deposition in the renal microvasculature (Figure 2) [64]. *APOL1* risk alleles were previously associated with ESRD in lupus nephritis patients of African descent [64], while *MYH9* is more relevant in European ancestry populations [65].

Environmental factors may also contribute to disease susceptibility. Chen et al. failed to identify modifying risk factors that can influence the *APOL1* high-risk genotype [34]. Similarly, previous studies did not succeed in associating the dietary acid load [66] and dietary patterns [67] with *APOL1* genotypes, potentially due to the need for larger study cohorts.

Genetic variations were also associated with CKD progression in the context of other underlying diseases. IgAN is an autoimmune disease characterized by mesangial proliferative glomerulonephritis associated with mostly IgA deposits, being the most common primary glomerulonephritis worldwide [22,68]. Many IgAN patients progress to ESRD within 20 years, but a small percentage experience rapid progression, suggesting genetic interference [22]. GWASs showed that a genetic IgAN risk score is associated with ESRD progression and strongly correlates with the East–West gradient in IgAN prevalence and with a North–South gradient within Europe [55,69]. Various genetic risk scores have been published, highlighting immune-related gene variants and IgA1 glycosylation, among others [25,69,70]. These variants contribute to the four processes that induce renal injury, aberrant IgA1 glycosylation, synthesis of antibodies directed against galactose-deficient IgA1, development of immune complexes, and accumulation of these complexes in the glomerular mesangium (Figure 2) [71]. Therefore, the use of multi-locus genetic risk scores might be promising for the prediction of disease susceptibility, and eventually, disease progression [69].

Diabetes is one of the most common underlying diseases associated with CKD, being also a significant risk factor for ESRD, particularly in the context of diabetic kidney disease [56]. The coexistence of the two diseases in patients represents a clinical challenge to manage glycemic control while preserving kidney function [72]. Genetic variations have a high influence on individuals’ vulnerability to diabetic kidney disease [73]. Accordingly, many studies included in this review studied different allele variations within the diabetic population. The SNPs in genes involved in oxidative stress (*SOD1/2* [28], *PPARG* [23], *GPX1* [33], *CYBA* [38], *MT2A* [31]), metabolism (*SPP1*, *BGLAP*, *MGP*, *CYP24A1* [24], *HSD11B1* [14], *ABCG8* [37]), RAAS [44], immune system (*ML2* [17]), transcription regulation (*EP300* [1]), and transmembrane channels (*AQP11* [30]), among others, were all related to an increased risk of CKD progression in the diabetic population. Chronic hyperglycemia results in progressive damage to the renal microvasculature that ultimately leads to compromised filtration and renal dysfunction [74]. Increased glucose levels induce inflammation and oxidative stress, causing the generation of reactive oxygen species (ROS) and immune cell infiltration in the renal tissue, with consequent lipid peroxidation, DNA damage, and fibrosis (Figure 2) [74].

Many studies also identified different gene variants that were associated with CKD independently of specific diseases. These allele variations may cause alterations in the structure and function of proteins that ultimately lead to renal tissue damage and CKD development and progression (Figure 2). Additionally, genetic variants in epigenetic modifiers can also contribute to the CKD/ESRD risk [26,27,41]. Nevertheless, many of these mechanisms remain to be elucidated, and further validation of such associations is needed.

Genetic testing in chronic kidney disease has the potential to enable more precise diagnosis, tailored disease monitoring and treatment, and family counseling [8]. However, there is a lack of evidence that the identification of such variations can improve clinical outcomes [8]. Enlarging sample sizes and studying cohorts with a more diverse ancestral composition enables the discovery of ancestry-specific effects and allows for a broader generalization of findings [8]. It is also essential to reveal the molecular and cellular mechanisms that lead to CKD in the context of a specific alteration. Identifying such mechanisms can promote the research and development of targeted therapies [75]. As an example, a recent phase-IIa study demonstrated that inaxaplin, a small-molecule compound that inhibits APOL1 function, reduced proteinuria in participants with two *APOL1* variants and focal segmental glomerulosclerosis [76].

The identification of genetic variants associated with CKD progression is challenging due to the frequent presence of comorbidities, the multiple causes of CKD, the multifactorial nature of the disease, and population-specific variations, which are not considered in all studies. The studies included in this systematic review differ in the design, cohort size, and CKD-related definitions. Not all the studies reported the allele frequency of the accessed SNPs, and many did not define the disease outcomes. The control definition was also variable among the studies, and not all of them recruited healthy individuals. The definition of CKD progression was also variable across the studies, while eGFR decline was used by some authors, but others used the creatinine levels or the time to initiate renal replacement therapy (development of ESRD).

## 5. Conclusions

This study showed that polymorphisms in different genomic regions may contribute to CKD progression. The described polymorphisms affect genes that encode proteins in different cellular pathways that ultimately promote kidney injury and dysfunction. Future studies with larger cohorts, stratified risk factors, and consistent definitions and outcomes are needed to support the described findings. The future elucidation of the mechanisms of validated associations may reveal new therapeutic targets and allow a personalized approach to the treatment of CKD.

## Figures and Tables

**Table 1:** Summary of the studies included in the systematic review.

Study	Study Type	n	Cause of CKD	Country	Genetic Variants
Fatumo, S., 2021 [12]	GWAS	11,512	na	Uganda and USA	GATM rs2433603 HBB rs141845179
Lee, F. Y., 2021 [13]	Cohort study, prospective	124	na	Malaysia	CYP3A5 rs776746
Mori, R. C., 2019 [14]	Cohort study, retrospective	466	T1D	Brazil	HSD11B1 rs11799634, rs17389016, rs846906, rs846910, rs4844880
Ahmad, N., 2020 [15]	Case-control, retrospective	600	T2D	Malaysia	NOS3 rs2070744 PPARGC1A rs8192678 KCNQ1 rs2283228, rs2237895
Adam, K. M., 2020 [16]	Case-control, retrospective	400	na	Kingdom of Saudi Arabia	UMOD rs12917707 MYH9 rs4821480
Cai, K., 2020 [17]	Cohort study, retrospective	210	T2D	China	MBL2 rs1800450, rs11003125, MBL2 rs7095891, rs7096206
Ibrahim, S. T., 2020 [18]	Case-control and cohort, retrospective	1237	na	UK	C3 R102G
Koo, B. K., 2020 [19]	Cohort study, prospective	396	NAFLD	Korea	MBOAT7-TMC4 rs626283 PNPLA3 rs738409 TM6SF2 rs5854292
Han, B., 2019 [20]	Cohort study, prospective	620	na	China	AQP11 rs2276415
Hessels, A. C., 2019 [21]	Cohort study, retrospective	241	AAV	The Netherlands	NR3C1 rs41423247, rs10052957, rs6190, rs6189, s6195, rs6198 HSD11B1 rs11119328
Ouyang, Y., 2019 [22]	Case-control, retrospective	1128	IgAN	China	MBL2 rs1800450, 7096206, rs7095891 FCN2 rs7851696, rs3124952, rs17514136
Satirapoj, B., 2019 [23]	Cohort study, prospective	422	T2D	Thailand	TCF7L2 rs7903146 PPARG2 Pro12Ala
Valls, J., 2019 [24]	Case-control, retrospective	3004	na	Spain	SPP1 rs1126616, rs9138 MMP3 rs35068180, rs679620 KL rs385564 CYP24A1 rs2248359 BGLAP rs1800247 OPG rs3102735; VDR rs2238135, rs731236 TNFRSF11B rs1564858 MGP rs4236
Shi, M., 2018 [25]	Cohort study, retrospective	1130	IgAN	China	HLA-DP rs1883414 HLA-DQ rs7763262, rs9275224 HLA-DQ/DR rs2856717 HLA-DR rs9275596 DEFA rs2738048, rs12716641, rs9314614, rs9314614 TAP2-PSMB9 rs2071543 HORMAD2 rs2412971 TNFSF13 rs3803800 VAV3 rs17019602 ITGAM-ITGAX rs11150612 ACCS rs2074038 KLF10/ODF1 rs2033562 ST6GAL1 rs7634389
Parsa, A., 2017 [26]	GWAS	3074	na	USA	LINC00923 rs653747 LINC00923 rs931891
Tang, K., 2017 [27]	Case-control, retrospective	1016	T2D	China	p300 rs20551 SIRT1 rs4746720
Chao, C. T., 2016 [28]	Case-control, prospective	1451	na	China	SOD2 rs4880 GPX1 rs1050450 PPAR-γ rs1801282, rs3856806
Guan, M., 2016 [29]	Case-control, retrospective	4218	T2D	USA	CD2AP/ADGRF2 rs116139597, rs11591277 MMP2 rs7185763 CLDN8 rs55884670 COL4A3 rs34505188 NPHP3-ACAD11 rs78174962 AC009495.3 rs6742727 ARHGAP24 rs10433935
Choma, D. P., 2016 [30]	Case-control, retrospective	978	T2D	USA	AQP11 rs2276415
Hattori, Y., 2016 [31]	Cohort study, retrospective	2774	na	Japan	MT2A rs28366003
Jiang, G., 2016 [32]	Cohort study, prospective	2755	T2D	China	G6PC2 rs478333 CDKAL1 rs7754840, rs7756992
Mohammedi, K., 2016 [33]	Cohort study, retrospective	1385	T1D	France and Belgium	GPX1 rs1987628, rs8179164, rs3448, rs9818758
Chen, T. K., 2015 [34]	Cohort study, retrospective	693	Hypertension	USA	APOL1 G1 rs73885319, rs60910145, rs71785313
Dai, C. S., 2015 [35]	Case-control, retrospective	331	na	Taiwan	HLA class I and II polymorphisms
Kelly, T. N., 2015 [36]	Cohort study, retrospective	2995	na	USA	REN HSD11B1 AGT AGTR1 NR3C2 CYP11B1 CYP11B2 HSD11B2 ACE ACE2 AGTR2 RENBP
Nicolas, A., 2015 [37]	Cohort study, retrospective	5277	T2D	France	ABCG8 rs11887534, rs4148217
Patente, T. A., 2015 [38]	Cohort study, retrospective	1357	T1D	France and Belgium	CYBA rs9932581, rs3794624, rs12709102, rs4673, rs1049255, rs11076692, 675T > A
Colares, V. S., 2014 [39]	Cohort study, retrospective	196	LN	Brazil	MYH9 rs4821480, rs2032487, rs4821481, rs3752462 APOL1 rs73885319, rs16996616, rs60910145, rs71785313 APOL3 rs11089781
Sandholm, N., 2013 [40]	GWAS	2668	T1D	Finland, UK, USA, Italy	chromosome 2q31.1 rs4972593
Ozdemir, O., 2014 [41]	Case-control, retrospective	440	na	Turkey	MTHFR C677T
Sambo, F., 2014 [42]	GWAS	7727	T1D	Finland, Denmark, UK, USA	WNT4/ZBTB40-rs12137135 RGMA/MCTP2-rs17709344 MAPRE1P2-rs1670754 SEMA6D/SLC24A5-rs12917114 SIK1-rs2838302
Oguri, M., 2013 [43]	Case-control study, prospective	1709	na	Japan	BTN2A1 rs6929846
Ilic, V., 2014 [44]	Cohort study, prospective	79	T1D	Serbia	AGT M235T ACE I/D AT1R A1166C
Tavira, B., 2013 [45]	Cohort study, retrospective	592	na	Spain	MYH9 rs3752462, rs4821480
Hubacek, J. A., 2012 [46]	Case-control, retrospective	10,354	na	Czech Republic	FTO rs17817449
Karsli Ceppioglu, S., 2011 [47]	Case-control, retrospective	124	na	Turkey	MGP T138C, Glu60X, Thr83Ala Klotho Cys370Ser
Corredor, Z., 2020 [48]	Case-control, retrospective	692	na	Spain	GPX1 rs17080528 GSTO1 rs2164624 GSTO2 rs156697 UMOD rs12917707 MGP rs4236
Lin, B. M., 2019 [49]	GWAS	41,041	na	USA	NMT2 rs10906850 APOL1 rs73885319, rs60910145 CDH8 rs11645800

AAV, anti-neutrophil cytoplasmic autoantibody (ANCA)-associated vasculitis. ACR, albumin/creatinine ratio. CKD, chronic kidney disease. DN, diabetic nephropathy. eGFR, estimated glomerular filtration rate. ESRD, end-stage renal disease. GC, glucocorticoid. GM, glomerulonephritis. GWAS, genome-wide association study. IgAN, immunoglobulin A nephropathy. LN, lupus nephritis. MDRD, modification of diet in renal disease. na, not applicable. RBC, red blood cell. RRT, renal replacement therapy. T1D, type-one diabetes. T2D, type-two diabetes. UAC, urinary albumin concentration. UACR, urine albumin–creatinine ratio. UAE, urinary albumin excretion. UK, United Kingdom. USA, United States of America. WHO, World Health Organization.

## Data Availability

Not applicable.

## Supplementary Materials

The following supporting information can be downloaded at https://www.mdpi.com/article/10.3390/biology14010068/s1, Table S1: Detailed summary of the studies included in this systematic review; Table S2: Risk of bias.
