# Supplementary material for: Genetic Variants Related to Increased CKD Progression—A Systematic Review
Source: Biology (Basel). 2025 Jan 14;14(1):68. doi: 10.3390/biology14010068 (PMC11761907; doi:10.3390/biology14010068)
Supplement: Supplementary file 1 [file biology-14-00068-s001.zip › biology-3320614-supplementary/Supplementary Table S1.pdf]

Supplementary Table S1: Detailed summary of studies included in this systematic review.

Study	Study type	n	Study Inclusion criteria	Study exclusion criteria	Country	Definitions	CKD progression (GFR decrease/unit)	Cause of CKD	Follow-up time	Outcomes	Genetic variants	Minor allele frequency
Fatumo, S. 2021	GWAS	Discovery cohort: 3288	• African individuals belonging to the nine ethnolinguistic groups from the Uganda GPC cohort	• Failed to meet the quality control for call rate<br>• Gender mismatch compared with X-chromosome	Uganda	• eGFR: CKD-EPI equation	na	na	na	na	• GATM rs2433603<br>• HBB rs141845179	GATM rs2433603: 48%<br>HBB rs141845179: 8%
		Validation cohort: 8224	• African American women from the WHI study (postmenopausal women aged 50-79 years)	na	USA	• eGFR: MDRD equation						
Lee, F. Y. 2021	Cohort study, prospective	124	• ≥ 18 years<br>• CKD patients with ≥ 30mL/min/1.73 m^2	• Pregnancy and lactation<br>• Incomplete medication regimen<br>• No routine nephrology care	Malaysia	• eGFR: CKD-EPI equation<br>• CKD and albuminuria: KDIGO 2012 guidelines	> 5mL/min/1.73m^2 / year	na	3 years	Rapid CKD progression: 23.4%	• CYP3A5 rs776746	23.8%
Mori, R.C. 2019	Cohort study, retrospective	466	• T1D patients with long-standing diabetes	na	Brazil	• eGFR: CKD-EPI equation<br>• DKD: Presence of macroalbuminuria (ACR > 300 mg/g) and/or reduced eGFR (< 60mL / min / 1.73 m^2)	≥ 5mL/min/1.73m^2 / year	T1D	10 years	Decliners of renal function: 29.1%	• HSD11B1 rs11799634<br>• HSD11B1 rs17389016<br>• HSD11B1 rs4844880<br>• HSD11B1 rs846910<br>• HSD11B1 rs846906	na

Ahmad, N. 2020	Case-control, retrospective	Cases: 300	• CKD patients<br>• T2D diagnosis	na	Malaysia	• eGFR: CKD-EPI equation<br>• CKD: <90 mL/min per 1.73 m^2 without microalbuminuria	≥25% decrease in estimated eGFR form baseline	T2D	Mean follow-up: 4.78 years	CKD progression: 13.3%	• NOS3 rs2070744<br>• PPARGC1A rs8192678<br>• KCNQ1 rs2237895<br>• KCNQ1 rs2283228	na
		Controls: 300	• Absence of CKD<br>• T2D diagnosis									
Adam, K.M. 2020	Case-control, retrospective	Cases: 154	• ESRD patients under haemodialysis for at least 3 months<br>• Absence of diabetes	• Diabetes<br>• Malignancy<br>• Infectious co-morbidities	Kingdom of Saudi Arabia	na	na	na	na	na	• UMOD rs12917707<br>• MYH9 rs4821480	• UMOD rs12917707: 12%<br>• MYH9 rs4821480: 23%
		Controls: 123	• Healthy individuals with normal renal function									• UMOD rs12917707: 13%<br>• MYH9 rs4821480: 8%
Cai, K. 2020	Cohort study, retrospective	Discovery cohort: 77	• T2D<br>• DN	• Other kidney diseases<br>• Active infections<br>• Severe liver dysfunction<br>• Under immunosuppressive therapy<br>• Kidney allograft recipients<br>• Reaching ESRD before renal biopsy<br>• Incomplete baseline data<br>• Lost follow-up	China	• eGFR: CKD-EPI equation<br>• T2D: American Diabetes Association 2017 proposed criteria<br>• DN: Renal Pathology Society 2010 proposed criteria<br>• ESRD: eGFR < 15 mL/min/1.73m2 or need of renal replacement therapy	na	T2D	Median follow-up: 28 months	ESRD development: 42.86%	• MBL2 rs1800450<br>• MBL2 rs11003125<br>• MBL2 rs7096206<br>• MBL2 rs7095891	na
		Validation cohort: 133							na	ESRD development: 43.61%		
Ibrahim, S. T., 2020	Study 1: Case-control, retrospective	Cases: 514	• ≥18 years<br>• eGFR <60 ml/min/1.73m2<br>• Absence of RRT	• <2 years follow-up<br>• <4 available eGFR values	UK	• eGFR: CKD-EPI equation<br>• ΔeGFR: linear regression (±ml/min/1.73m^2/y	• Rapid CKD progressors: ΔeGFR ≤ 3ml/min/1.73m^2/yr	na	56 months median follow-up	• CKD progression: 91.9% for rapid progressors,	C3 R102G	25.7%

		Control s: 454	• Ethnically matched individuals<br>• Unrelated from controls<br>• Healthy	na		r)<br>• ESRD: RRT or eGFR<10 ml/min/1.73m^2	•CKD progression: ESRD or ≥ 40% eGFR decline			9% for stable progressors<br>• Death: 13.9% for rapid progressors, 22.7% for stable progressors		20.6%
	Study 2: Cohort study, retrosp ective	Study 2: 269	• ≥18 years<br>• eGFR <60 ml/min/1.73m2<br>• Absence of RRT<br>• Biopsy proven GN, regardless of their ΔeGFR<br>• Available DNA samples	Renal diagnosis other than DN					na	na		23.4%
Koo, B. K., 2020	Cohort study, prospec tive	396	• ≥18 years<br>• Increased liver/kidney echogenicity and posterior attenuation on bright echogenic liver on ultrasound scanning<br>• Unexplained high alanine aminotransferase levels above the reference range within the previous 6 months<br>• Liver biopsy	• Hepatitis B or C<br>• Autoimmune hepatitis<br>• Primary biliary cholangitis<br>• Primary sclerosing cholangitis<br>• Drug-induced liver injury or steatosis<br>• Wilson disease or hemochromatosis<br>• Excessive alcohol consumption (male >30 g/day; female >20 g/day)<br>• Diagnosis of malignancy within the previous year	Korea	• eGFR: MDRD formula<br>• BMI: World Health Organization Asia–Pacific criteria<br>• Metabolic syndrome: revised National Cholesterol Education Program Adult Treatment Panel III criteria<br>• NAFLD: presence of ≥5% macrovesicular steatosis<br>• Non-alcoholic steatohepatitis: Brunt et al. criteria<br>• Fibrosis: 5-point scale by Brunt and modified by Kleiner et al.	na	NAFL D	na	na	• MBOAT7-TMC4 rs626283<br>• PNPLA3 rs738409<br>• TM6SF2 rs5854292	na

Han, B., 2019	Cohort study, prospective	620	• ≥18 and ≤ 80 years<br>• Chinese ethnicity<br>• CKD diagnosis (stages 2–5)<br>• Written informed consent	• Dialysis<br>• Kidney transplantation<br>• Malignancy<br>• Severe hypercreatinemia	China	• CKD: eGFR <90mL/min/1.73m^2 for 3 months, using the 4-variable MDMR formula<br>• Severe hypercreatinemia: creatinine 177 mmol/L for men, 159 mmol/L for women	Increase in creatinine levels of 0.4mg/dL (35 mmol/L) or more above baseline, maintenance dialysis initiation or transplantation.	na	3 years	CKD progression: 27.4%	AQP11 rs2276415	na
Hessels, A. C., 2019	Cohort study, retrospective	241	Treatment with GCs combined with another immunosuppressive drug as initial treatment, according to the EULAR/ European Renal Association European Dialysis and Transplant Association recommendations	Eosinophilic granulomatosis with polyangiitis (formerly Churg Strauss syndrome)	The Netherlands	na	na	AAV	10 years	• Relapse-free survival: 92% (1 year), 53% (5 years), 38% (10 years)<br>• Cumulative survival: 97% (1 year), 88% (5 years), 72% (10 years)<br>• ESRD: 11% (10 years)	• NR3C1 rs41423247<br>• NR3C1 rs10052957<br>• NR3C1 rs6198<br>• NR3C1 s6195<br>• NR3C1 rs6189<br>• NR3C1 rs6190<br>• HSD11B1 rs11119328	• NR3C1 rs41423247: 40%<br>• NR3C1 rs10052957: 32%<br>• NR3C1 rs6198: 17%<br>• NR3C1 s6195: 5%<br>• NR3C1 rs6189: 4%<br>• NR3C1 rs6190: 4%<br>• HSD11B1 rs11119328: 19%
Ouyang, Y., 2019	Case-control, retrospective	Discovery cohort: 606 cases	• IgAN<br>• eGFR ≥15 mL/min/1.73 m^2 at diagnosis<br>• Minimum follow-up of 12-months<br>• DNA sample available<br>• Signed informed consent	• Henoch-Schoenlein purpura<br>• Systemic lupus erythematosus<br>• Liver disease	China	• eGFR: CKD-EPI equation<br>• ESRD: eGFR <15 ml/min/1.73 m^2 or need for dialysis/renal transplantation<br>• IgAN: Renal biopsy demonstrating dominant IgA deposition in the mesangium of glomeruli by immunofluorescence microscopy<br>• CKD: K/DOQI	ESRD	IgAN	Median follow-up: 40.7 months	13.7%	• MBL2: rs1800450<br>• MBL2: 7096206<br>• MBL2: rs7095891<br>• FCN2: rs3124952<br>• FCN2: rs17514136<br>• FCN2: rs7851696	na
		Validation cohort: 401 cases							Median follow-up: 53.9 months	14.7%		
		Controls: 121	Healthy individuals	na					na	na		

						practice guidelines<br>• IgAN: CLIN-PATH equation						
Satirapoj, B., 2019	Cohort study, prospective	422	• ≥18 years<br>• T2DM	• Active malignancy<br>• Severe heart, lung, or liver disease<br>• Previous stroke<br>• Chronic infection within 1 year of starting the study<br>• Any immunological or inflammatory disorders	Thailand	• T2DM: 2015 American Diabetes Association diagnostic criteria<br>• eGFR: 2009 CKD-EPI creatinine equation<br>• CKD: eGFR < 60 mL/min/1.73 m^2<br>• ESRD: eGFR < 15 mL/min/ 1.73 m2 or need for long-term renal replacement therapy<br>• Cardiovascular events: New onset of acute coronary syndrome, myocardial infarction, unstable angina, or new cardiac procedure<br>• Cerebrovascular events: new onset of transient ischemic attack, cerebral ischemia, or cerebral hemorrhage	na	T2D	5 years	• Cardiovascular events: 15.9%<br>• Cerebrovascular events: 8.8%<br>Death: 8.3%	• TCF7L2 rs7903146<br>• PPARG2 Pro12Ala	na
Valls, J., 2019	Case control, retrospective	Cases: 2445<br>Controls: 559	Cases: • ≥18 and ≤ 74 years<br>• CKD stage ≥3<br>Controls: MDRD4 > 60 mL/min/1.73 m^2	• Pregnancy<br>• Active infections<br>• Life expectancy <12 months<br>• Previous cardiovascular event<br>• Carotid artery surgery<br>• Any organ transplantation	Spain	• eGFR: MDRD formula<br>• CKD: eGFR < 60 mL/min/1.73 m^2	na	na	na	na	• SPP1 rs1126616<br>• MMP3 rs35068180<br>• VDR rs2238135<br>• OPG rs3102735;<br>• BGLAP rs1800247<br>• KL rs385564<br>• MMP3 rs679620	na

											• CYP24A1 rs2248359<br>• TNFRSF11B rs1564858<br>• MGP rs4236<br>• SPP1 rs9138<br>• VDR rs731236	
Shi, M., 2018	Cohort study, retrospective	Discovery cohort: 613<br>Validation cohort: 517	• ≥18 years<br>• Follow-up time of ≥12 months<br>• Available baseline data	• eGFR ≥15 ml/min per 1.73 m² at the time of biopsy<br>• Alport syndrome<br>• Thin basement membrane disease<br>• SLE<br>• Henoch–Schonlein purpura<br>• Other systemic diseases.	China	• eGFR: CKD-EPI equation<br>• IgAN: defined by kidney biopsy., with histologic changes were scored using the Oxford-MESTC score<br>• Primary outcome: ESRD and a 50% reduction of eGFR from baseline	na	IgAN	Median follow-up: 50 months	Primary outcome: 19%	• HLA-DP rs1883414<br>• HLA-DQ rs7763262<br>• HLA-DQ rs9275224<br>• HLA-DQ/DR rs2856717<br>• HLA-DR rs9275596<br>• DEFA rs2738048<br>• DEFA rs12716641<br>• DEFA rs9314614<br>• TAP2-PSMB9 rs2071543<br>• HORMAD2 rs2412971<br>• TNFSF13 rs3803800<br>• VAV3 rs17019602<br>• ITGAM-ITGAX rs11150612<br>• ACCS rs2074038<br>• KLF10/ODF1 rs2033562<br>• ST6GAL1 rs7634389	na

Parsa, A., 2017	GWAS	3074	• ≥ 21 and ≤ 74 years<br>• eGFR ≥ 20 and ≤ 70 ml/min per 1.73 m^2.	• GN requiring immunosuppressive therapy<br>• Advanced heart failure<br>• Cirrhosis<br>• Polycystic kidney disease	USA	• eGFR: estimated from a previously developed and validated equation on the basis of measures from the CRIC Study participants<br>• Diabetes: fasting glucose ≥126 mg/dl, a non-fasting glucose ≥200 mg/dl, or use of insulin or an oral hypoglycaemic agent<br>• eGFR slope: linear model of multiple eGFR values per person measured annually	na	na	4 years	na	• LINC00923 rs653747<br>• LINC00923 rs931891	LINC00923 rs653747: 47%
Tang, K., 2017	Case-control, retrospective	628 Cases	• T2D<br>• DKD	• Any severe infection<br>• Serious trauma during the previous 3 months<br>• Malignancy<br>• Autoimmune diseases<br>• Critical heart failure<br>• Severe kidney injury<br>• Liver dysfunction<br>• Lack of diabetic retinopathy<br>• Low or quickly declining GFR<br>• Nephritic syndrome or rapidly rising proteinuria<br>• Refractory hypertension<br>• Active urinary	China	• eGFR: CKD-EPI equation<br>• Hypertension: blood pressure ≤ 140/90 mmHg	na	T2D	na	na	• p300 rs20551<br>• SIRT1 rs4746720	• p300 rs20551: 12.10%<br>• SIRT1 rs4746720: 46.34%
		388 Controls	• T2D									• p300 rs20551: 7.86%<br>• SIRT1 rs4746720: 48.324%

				sediment<br>• > 30 % GFR reduction in 2 to 3 months after commencement of an ARB or ACE inhibitor if the k-idney damage was not linked to the presence of diabetes								
Chao, C. T., 2016	Multicentre, observational, prospective	671 Cases	ESRD patients who initiated long-term haemodialysis or peritoneal dialysis	na	China	• Diabetes: history of physician-diagnosed diabetes or 2 separate episodes of fasting blood glucose levels ≥126mg/dL (7.0 mmole/L).<br>• Diabetic nephropathy as the cause of ESRD: Patients who had a long-standing diabetes history (at least 5 years) before dialysis start, presence of diabetic retinopathy, and/or proteinuria (≥100mg/dL by urinalysis) without other causes.	na	na	na	na	• SOD2 rs4880<br>• GPX1 rs1050450<br>• PPAR-γ rs1801282<br>• PPAR-γ rs3856806	• SOD2 rs4880: 17.36%<br>• GPX1 rs1050450: 5.29%<br>• PPAR-γ rs1801282: 4.99%<br>• PPAR-γ rs3856806: 26.53%
		780 Controls	No CKD									• SOD2 rs4880: 13,97%<br>• GPX1 rs1050450: 5.38%<br>• PPAR-γ rs1801282: 3.08%<br>• PPAR-γ rs3856806: 24.29%
Guan, M., 2016	Case-control, retrospective	Cases: 2524	• T2D<br>• ESRD	ESRD attributed to non-diabetic causes: surgical or urologic causes, polycystic kidney disease, autoimmune disease, hepatitis, IgA nephropathy, membranous glomerulonephritis, membranoprolifer	USA	T2D-ESRD: Diagnosis of diabetes > 25 years and for ≥5 years prior to the onset of ESRD (or in the presence of diabetic retinopathy ensuring adequate T2D durations), and one or more of the following:	na	T2D	na	na	• CD2AP/ADGRF2 rs116139597<br>• CD2AP/ADGRF2 rs11591277<br>• MMP2 rs7185763<br>• CLDN8 rs55884670<br>• COL4A3	na

				ative glomerulonephritis, or monogenic kidney diseases		renal replacement therapy, eGFR <30 ml/min/1.73 m2 (N = 43), or UACR >300 mg/g, when eGFR was missing.					rs34505188 • NPHP3-ACAD11 rs78174962 • AC009495.3 rs6742727 • ARHGAP24 rs10433935	
		Controls: 1694	• No nephropathy (eGFR ≥60 ml/min/1.73 m^2 + UACR <30 mg/g)	na				na				
Choma, D. P., 2016	Retrospective case-control studies	Cases: 295	• ≥18 years • Caucasian • Follow-up ≥5 and ≤10 years • ≥5 outpatient eGFR during follow-up	• eGFR <15 ml·min-1 ·1.73 m-22; • Diagnosis of CKD stage 5 • ESRD at baseline • Current or prior kidney malignancy • Kidney transplant • Pregnancy	USA	• eGFR: MDRD formula • CKD: at least two eGFR values of <50 ml·min-1 ·1.73 m^{-2} • Cases: Patients who experienced AKI or CKD • Primary outcome: development of AKI (0.3– 0.5 mg/dl or a 25–50% increase of serum creatinine from a baseline level)	na	T2D	10 years	na	AQP11 rs2276415	na
		Controls: 683	• Patients matching the case by sex and age within 5 years • No AKI or CKD • No serum or plasma creatinine values of ≤1 mg/dl.	• Absence of any case definitions for AKI or CKD • Average eGFR calculated from creatinine data of >70 ml·min-1 ·1.73 m^{-2} • No episodes of eGFR <60 ml·min-1 ·1.73 m^{-2} during follow-up • No outpatient or inpatient ICD-9 codes for an acute or chronic kidney condition • No serum or plasma creatinine values of >1 mg/dl.								

Hattori, Y., 2016	Cohort study, retrospective	2774	• ≥35 to ≤ 69 years<br>• Residents in Nagoya City with a certificate of residence	Foreign resident registrations	Japan	na	na	na	5 years	na	MT2A rs28366003	na
Jiang, G., 2016	Cohort study, prospective	2755	T2D	• CKD diagnosis at enrolment<br>• non-Chinese or unknown ethnicity	China	• eGFR: Chinese-modified MDRD<br>• Microalbuminuria: ACR ≥2.5 mg/mmol and ≥3.5 mg/mmol for men and women, respectively<br>• CKD: fatal and nonfatal diabetes with renal manifestations, chronic kidney disease, or unspecified renal failure; or dialysis; or peritoneal dialysis; or eGFR < 60 ml/min per 1.73 m2 during follow-up period<br>• ESRD: dialysis or peritoneal dialysis or eGFR < 15 ml/min per 1.73 m2.	na	T2D	Median follow-up: 7.7 years	na	• G6PC2 rs478333<br>• CDKAL1 rs7754840<br>• CDKAL1 rs7756992	na

Mohammedi, K., 2016	Cohort study, retrospective	1385	T1D	na	France and Belgium	• eGFR: MDRD formula<br>• ESRD: haemodialysis or kidney transplantation<br>• No nephropathy: UAC <30 mg/24 h or <20 μg/min or <20 mg/L and plasma creatinine <150 μmol/L in at least 2 of 3 consecutive assessments<br>• Incipient nephropathy: UAC = 30–300 mg/24 h or 20–200 μg/min or 20–200 mg/L and plasma creatinine <150 μmol/L<br>• Established nephropathy: UAC >300 mg/24 h or >200 μg/min or >200 mg/L and plasma creatinine <150 μmol/L<br>• Advanced nephropathy: past or present macroalbuminuria and plasma creatinine >150 μmol/L or renal replacement therapy	na	T1D	10 years	na	• GPX1 rs1987628<br>• GPX1 rs8179164<br>• GPX1 rs3448<br>• GPX1 rs9818758	na

Chen, T. K., 2015	Cohort study, retrospective	693	• ≥18 and ≤ 70 years • Self-identified African Americans • CKD attributed to hypertension • eGFR between 20 and 65 ml/min/1.73m	• Cause for CKD other than hypertension • DM or fasting blood glucose level > 140 mg/dl • Urine protein/creatinine ratio >2.5g/g • Secondary hypertension • Accelerated/malignant hypertension in the previous 6 months.	USA	• Hypertension: diastolic blood pressure > 95 mmHg • ESRD: dialysis or renal transplantation • Primary outcome: time from baseline to CKD	Doubling of serum creatinine from baseline or the development of ESRD	Hypertension	Mean follow-up: 7.8 years	CDK progression: 58% (APOL1 high–risk group) and 37% (APOL1 low–risk group)	• APOL1 G1 rs73885319 • APOL1 G1 rs60910145 • APOL1 G2 rs71785313	na
Dai, C. S., 2015	Case-control, retrospective	Cases: 141	• <50 years • ESRD • Awaiting kidney transplantation between 2002 and 2013 at the Keelung branch of Chang Gung Memorial Hospital	na	Taiwan	na	na	na	na	na	HLA class I and II polymorphisms	na
		Controls: 190	Healthy individuals									
Kelly, T. N., 2015	Cohort study, retrospective	2995	• ≥21 and ≤74 years • Mild-to-moderate CKD • Adequate phenotype and genotype data	• Institutionalized (e.g., prisoner, nursing home or skilled nursing facility resident) • Unable or unwilling to give informed consent • Unlikely or unable to participate in required study procedures • New York Heart Association class III or IV heart failure (baseline) • Cirrhosis	USA	• eGFR: CRIC-specific equation • CKD: eGFR 20–70 mL/min/1.73 m^2 • ESRD: dialysis or kidney transplant	Incident ESRD or halving of eGFR from baseline	na	Median follow-up: Whites: 3.7 years Blacks: 3.5 years	• Renal events: 12% of whites and 25% of blacks • eGFR decline: average of 1.2 mL/min/1.73 m2/year in whites and 2.3 mL/min/1.73 m2/year in blacks	• REN • HSD11B1 • AGT • AGTR1 • NR3C2 • CYP11B1 • CYP11B2 • HSD11B2 • ACE • ACE2 • AGTR2 • RENBP	na

				• HIV infection and/or AIDS<br>• Pregnancy<br>• Previous dialysis<br>• Previous organ or bone marrow transplant<br>• Immunosuppressive or other immunotherapy for primary renal disease or systemic vasculitis within the previous 6 months<br>• Previous diagnosis of multiple myeloma or renal carcinoma<br>• Polycystic kidney disease<br>• Current participation in interventional clinical trial or in a research study								
Nicolas, A., 2015	Cohort study, retrospective	Discovery cohort: 3137	• ≤ 50 years<br>• T2D<br>• Treatment with oral antidiabetic drugs (insulin excluded)<br>• High urinary albumin levels (76% micro-, 24% macro-albuminuria patients)<br>• Serum creatinine concentrations ≤150 µmol/l (1.7 mg/dl)	• Serum creatinine concentration > 150µmol/l<br>• Treatment with insulin, ACE inhibitors, or angiotensin II receptor blockers<br>• Documented congestive chronic heart failure<br>• Myocardial infarction in the previous t3 months<br>• Urinary tract infection<br>• Previous intolerance to ACE inhibitors	France	• Renal events: doubling of serum creatinine concentration or ESRD<br>• ESRD: requirement for dialysis or kidney transplant	na	T2D	Mean follow-up: 4 years	Renal events: 75 renal events (66 doublings of serum creatinine concentration and 9 cases of ESRD)	• ABCG8 rs11887534<br>• ABCG8 rs4148217	ABCG8 rs11887534<br>• Patients with renal event: 3.4%<br>• Patients without renal event: 6.6%<br>ABCG8 rs4148217<br>• Patients with renal event: 28%<br>• Patients without renal event: 19.2%

		Validation cohort: 2140	• Diabetic nephropathy in T2D<br>• T2D patients with high urinary albumin concentrations (>20 mg/l or 30 mg/24 h on two of three sterile urine collections)<br>• Retinopathy	na					na	na		ABCG8 rs4148217<br>• Patients with ESRD: 19.5%<br>• Patients without ESRD: 27.3%
Patente, T. A., 2015	Cohort study, retrospective	Cohort 1: 340	• <40 years at the onset of diabetes<br>• T1D for ≥3 years<br>• No chronic disease unrelated to diabetes<br>• Any stage of retinopathy or nephropathy (except renal replacement therapy)	• Nondiabetic renal disease	France	• No nephropathy: UAE <30 mg/24 h or <20 mg/min or <20 mg/L and plasma creatinine <150 mmol/L in at least two of three consecutive assessments<br>• Incipient nephropathy: persistent microalbuminuria (UAE = 30–300 mg/24 h or 20–200 mg/min or 20–200 mg/L) and plasma creatinine <150 mmol/L in at least two of three consecutive assessments<br>• Established nephropathy: past or present macroalbuminuria (UAE 4300 mg/24 h or 4200 mg/min or 4200 mg/L) and plasma creatinine <150 mmol/L<br>• Advanced nephropathy: past or present macroalbuminuria	na	T1D	Mean follow-up: 10 years	Renal event: 28.8%	• CYBA rs9932581<br>• CYBA rs3794624<br>• CYBA rs4673<br>• CYBA rs12709102<br>• CYBA rs1049255<br>• CYBA rs11076692<br>• CYBA 675T>A	Renal event<br>rs9932581: 43.3%, rs3794624: 34.0%, rs4673: 35.1%, rs12709102: 39.5%, rs1049255: 45.7%, rs11076692: 48.0%, 675T>A: 7.2%<br>No renal event<br>rs9932581: 41.0%, rs3794624: 31.7%, rs4673: 30.1%, rs12709102: 39.9%, rs1049255: 47.1%, rs11076692: 48.6%, 675T>A: 6.6%
		Cohort 2: 444	• T1D before the age of 35 years<br>• Past or present proliferative diabetic retinopathy	• Terminal cancer<br>• Personal disability	France and Belgium				Mean follow-up: 9 years	ESRD: 11.4%		No nephropathy<br>rs9932581: 35.2%, rs3794624: 37.1%, rs4673: 29.7%, rs12709102: 42.1%, rs1049255: 47.0%, rs11076692: 50.0%, 675T>A: 9.1%<br>Incipient nephropathy<br>rs9932581: 41.1%, rs3794624: 29.4%, rs4673: 33.5%, rs12709102: 42.3%, rs1049255: 48.7%, rs11076692: 49.0%, 675T>A: 6.6%<br>Established and advanced
		Cohort 3: 573	T1D	na					Mean follow-up: 6 years	ESRD: 5.5%		

						and plasma creatinine 4150 mmol/L or renal replacement therapy<br>• Retinopathy: staged according to Kohner's classification<br>• eGFR: MDRD formula<br>• Renal event: new case of microalbuminuria or the progression to a more severe stage of nephropathy<br>• ESRD: requirement of renal replacement therapy (dialysis or kidney transplantation).<br>• Arterial hypertension: use of antihypertensive drugs and previous history of hypertension.					nephropathy<br>rs9932581: 36.1%,<br>rs3794624: 30.9%,<br>rs4673: 34.2%,<br>rs12709102: 41.8%,<br>rs1049255: 49.6%,<br>rs11076692: 51.1%,<br>675T>A: 7.6%

Colares, V. S., 2014	Cohort study, retrospective	196	• ≥18 years<br>• Renal biopsy confirmed diagnosis of LN<br>• Underwent regular follow-up between July 2005 and July 2007	• Diabetes<br>• Hepatitis B and C<br>• HIV<br>• Less than 3 months of follow-up	Brazil	•LN: WHO classification<br>• Renal flare: (i) recurrence or the development of nephrotic syndrome (serum albumin ≤3.5 g/dl and 24 h proteinuria ≥3 g; (ii) renal impairment (≥33% increase of serum creatinine within a 1-month period directly attributed to lupus and confirmed 1 week later; flare referred to as 'nephritic flare') or (iii) a threefold increase of 24 h proteinuria within a 3-month period accompanied by microscopic haematuria<br>•Microscopic haematuria: number of RBC per high power field superior to upper normal limit for the local laboratory and ≥33% reduction of serum C3 level within a 3-month period, only to those patients with low grade baseline 24 h proteinuria (≥0.5 g and <1 g)<br>•Renal remission: serum creatinine ≤1.4 mg/dl and a	na	LN	Mean follow-up: 6.2 years	na	• MYH9 rs4821480<br>• MYH9 rs2032487<br>• MYH9 rs4821481<br>• MYH9 rs3752462<br>• APOL1 rs73885319<br>• APOL1 rs16996616<br>• APOL1 rs60910145<br>• APOL1 rs71785313<br>• APOL3 rs11089781	na

						24 h proteinuria <0.3 g and a urinary RBC count ,10/ high power field, at any time of follow-up<br>•Partial remission: 24 h proteinuria between 0.3 and 2.9 g, or a proteinuria drop >50% from the baseline, at any time of follow-up.<br>•Primary outcome: duplication of serum creatinine or need of renal replacement therapy						
Sandholm, N., 2013	GWAS	Discovery cohort: 2235	T1D	T1D patients who were known to have ESRD due to any nondiabetic cause	Finland	ESRD: need for dialysis or kidney transplant	na	T1D	na	na	chromosome 2q31.1 rs4972593	Women<br>ESRD: 17%<br>non-ESRD: 9%<br>Men<br>ESRD: 11%<br>Non-ESRD: 11%
		Validation cohort: 433			UK, USA, Italy							Women<br>ESRD<br>(UK/USA/Italy): 18/17/18%<br>Non-ESRD<br>(UK/USA/Italy): 16/13/15%<br>Men<br>ESRD<br>(UK/USA/Italy): 16/14/14%<br>Non-ESRD<br>(UK/USA/Italy): 14/13/17%
Ozdemir, O., 2014	Case-control, retrospective	Cases: 228	ESRD patients requiring long-term haemodialysis	na	Turkey	na	na	na	na	na	MTHFR C677T	Cases: 28.1%

		Controls: 212	Heathy individuals from the same ethnicity									Controls: 13.0
Sambo, F., 2014	GWAS	Discovery cohort: 3464	• T1D<br>• Age at onset of diabetes ≤40 years<br>• Time to definite insulin therapy ≤1 year before diagnosis	na	Finland	• DN: persistent macroalbuminuria (urinary AER ≥200 µg/min [overnight urine collection] or ≥300 mg/24 h [24 h urine collection], or a urinary ACR ≥25 mg/mmol for men and ≥35 mg/mmol for women, or dipstick ≥1) in 2 out of 3 consecutive measurements and an absence of other known kidney or urinary tract diseases<br>• Microalbuminuria: 20≤AER<200 µg/min or 30≤AER<300 mg/24 h or 2.5≤ACR<25 mg/mmol for men and 3.5≤ACR<35 mg/mmol for women in two out of three consecutive urine collections<br>• Absence of nephropathy was defined as a persistent normal AER (AER<20 µg/min or <30 mg/24 h, or ACR<2.5 mg/mmol for men or <3.5 mg/mmol for	na	T1D	na	na	• WNT4/ZBTB40-rs12137135<br>• RGMA/MCTP2-rs17709344<br>• MAPRE1P2-rs1670754<br>• SEMA6D/SLC24A5-rs12917114<br>• SIK1-rs2838302	• WNT4/ZBTB40 rs12137135: 16%<br>• RGMA/MCTP2 rs17709344: 2%<br>• MAPRE1P2-rs1670754: 15%<br>• SEMA6D/SLC24A5 rs12917114: 15%<br>• SIK1 rs2838302: 8%
		Validation cohort: 4263	T1D		Denmark, UK, USA							• WNT4/ZBTB40 (Italy, UK, USA): 18/15/9%<br>• RGMA/MCTP2 rs17709344 (Italy, UK, USA): 4/3/2%<br>• MAPRE1P2 rs1670754 (Italy, UK, USA): 21/24/16%<br>• SEMA6D/SLC24A5 rs12917114 (Italy, UK, USA): 10/10/8%<br>• SIK1 rs2838302 (Italy, UK, USA): 7/9/8%

						women), after at least 15 years of diabetes<br>• ESRD: ongoing dialysis treatment or a past kidney transplant						
Oguri, M., 2013	Case-control study, prospective	Cases: 435	eGFR is <60 ml/min per 1.73 m2<br>Controls	na	Japan	• eGFR: MDRD equation<br>• CKD: eGFR is <60 ml/min per 1.73 m²	na	na	na	na	BTN2A1 rs6929846	na
		Controls: 1274	eGFR was ≥90 ml/min per 1.73 m2	Renal disease or major health problems								
Ilic, V., 2014	Cohort study, prospective	79	• ≥20 to ≤40 years<br>• T1D<br>• Duration of diabetes > 5 years<br>• Absence of other severe diseases<br>• Hospitalised in the Institute of Diabetes and Metabolic Diseases from 2008 to 2010.	Severe diseases	Serbia	• Normoalbuminuric: UAE < 30 mg/24 h<br>• Microalbuminuria: UAE 30–300 mg/24 h<br>• Macroalbuminuria or manifested proteinuria over 300 mg/24 h	na	T1D	na	na	• AGT M235T<br>• ACE I/D<br>• AT1R A1166C	• AGT M235T (Normoalbuminuric/ Microalbuminuria/Proteinuria): 44/52.4/62%<br>• ACE I/D (Normoalbuminuric/ Microalbuminuria/Proteinuria): 48.5/40.5/52%<br>• AT1R A1166C (Normoalbuminuric/ Microalbuminuria/Proteinuria): 29/33/34
Tavira, B., 2013	Cohort study, retrospective	592	• ≥55 to ≤85 years<br>• Caucasian<br>• non-related participants Residents in Asturias	na	Spain	• eGFR: MDRD) formula<br>• CKD: presence of renal impairment with an eGFR ‹ 60	na	na	na	na	• MYH9 rs3752462<br>• MYH9 rs4821480	• MYH9 rs3752462: 31%<br>• MYH9 rs4821480: 8%

Hubacek, J. A., 2012	Study 1: Case-control, retrospective	Cases: 984	• ESRD<br>• Dialysis therapy for at least 3 months	• Generalized cancer<br>• History of poisoning<br>• Another known exogenous cause of ESRD	Czech Republic	na	na	na	na	na	FTO rs17817449	na
		Controls: 2501	Participants in the Czech post-MONICA study	na								
	Study 2: Case-control, retrospective	Cases: 188	Patients who underwent kidney transplantation for CKD/ESRD at the Institute of Clinical and Experimental Medicine in Prague in 1999–2007.	• Generalized cancer<br>• History of poisoning<br>• Another known exogenous cause of ESRD								
		Controls: 6681	Participants in the Czech HAPIEE study	na								
Karsli Ceppioglu, S., 2011	Case-control, retrospective	Cases: 84	CKD	Malignant tumours	Turkey	CKD: KDOQI 2009 guidelines (patients with 15 < glomerular filtration rate ≥ 59 mL/min/1.73 m2 were considered as stage 3–4 CKD)	na	na	na	na	• MGP T138C<br>• MGP Glu60X<br>• MGP Thr83Ala<br>• Klotho Cys370Ser	na
		Controls: 37	Healthy individuals									
Corredor, Z., 2020	Case-control, retrospective	Cases: 548	eGFR < 60 mL/min/1.73 m2	na	Spain	na	na	na	7 years	na	• GPX1 rs17080528<br>• GSTO1 rs2164624<br>• GSTO2 rs156697	na

		Controls: 174	• Suffering from either prostatic pathology, urinary tract infections or kidney stones<br>• Normal eGFR, according to age								• UMOD rs12917707<br>• MGP rs4236	
Lin, B. M., 2019	GWAS	41041	Kidney phenotype information	European ancestry	USA	• eGFR: CKD-EPI equation<br>CKD: eGFR ≥15 and ≤60 ml/min/1.73 m^2<br>• ESRD: eGFR< 15 ml/min/1.73m^2	na	na	na	na	• NMT2 rs10906850<br>• APOL1 rs73885319<br>• APOL1 rs60910145<br>• CDH8 rs11645800	• NMT2 rs10906850: 22.5%<br>• APOL1 rs73885319: 7.7%<br>• CDH8 rs11645800: 18.4%

AAV, Anti-neutrophil cytoplasmic autoantibody (ANCA)-associated vasculitis. ACR, albumin/creatinine ratio. CKD, chronic kidney disease. DN, diabetic nephropathy. eGFR, Estimated Glomerular Filtration Rate. ESRD, end-stage renal disease. GC, glucocorticoid. GM, glomerulonephritis. GWAS, genome-wide association study. IgAN, immunoglobulin A nephropathy. LN, Lupus nephritis. MDRD, modification of diet in renal disease. na, not applicable. RBC, red blood cells. RRT, renal replacement therapy. T1D, type one diabetes. T2D, type two diabetes. UAC, urinary albumin concentration. UACR, urine albumin-creatinine ratio. UAE, urinary albumin excretion. UK, United Kingdom. USA, United States of America. WHO, World Health Organization.
