# Supplementary material for: Genetic Variants Related to Increased CKD Progression—A Systematic Review
Source: Biology (Basel). 2025 Jan 14;14(1):68. doi: 10.3390/biology14010068 (PMC11761907; doi:10.3390/biology14010068)
Supplement: Supplementary file 1 [file biology-14-00068-s001.zip › biology-3320614-supplementary/Supplementary Table S2.pdf]

Supplementary Table S2: Risk of bias.

	Selection				Comparability	Exposure/Outcome			
**Study identification**	**Is the case definition adequate?**	**Representativeness of the cases/exposed cohort**	**Selection of controls/non-exposed cohort**	**Definition of controls**	**CKD progression (eGFR)**	**Assessment of the outcome/exposure**	**Same method for cases and controls**	**non-response rate**	**Quality**
Fatumo, S. 2021	*	*	*	*	*		*	*	Medium
Lee, F. Y. 2021	*	*	*	*	**	*	*	*	High
Mori, R.C. 2019	*	*	*	*	**	*	*	*	High
Ahmad, N. 2020	*	*	*	*	**		*	*	High
Adam, K.M. 2020	*		*	*	*		*	*	Medium

Cai, K. 2020	*		*	*	*	*	*	*	High
Ibrahim, S. T., 2020	*	*	*	*	**	*	*	*	High
Koo, B. K., 2020	*	*	*	*	*		*	*	Medium
Han, B., 2019	*	*	*	*	**	*	*	*	High
Hessels, A. C., 2019	*	*	*	*	*	*	*	*	High
Ouyang, Y., 2019	*	*	*	*	**	*	*	*	High
Satirapoj, B., 2019	*	*	*	*	*	*	*	*	High
Valls, J., 2019	*	*	*	*	*		*	*	Medium
Shi, M., 2018	*	*	*	*	*	*	*	*	High
Parsa, A., 2017	*	*	*	*	*		*	*	Medium
Tang, K., 2017	*	*	*	*	*		*	*	Medium

Chao, C. T., 2016	*	*	*	*	*		*	*	Medium
Guan, M., 2016	*	*	*	*	*		*	*	Medium
Choma, D. P., 2016	*	*	*	*	*		*	*	Medium
Hattori, Y., 2016	*	*	*	*	*		*	*	Medium
Jiang, G., 2016	*	*	*	*	*		*	*	Medium
Mohammedi, K., 2016	*	*	*	*	*		*	*	Medium
Chen, T. K., 2015	*	*	*	*	**	*	*	*	High
Dai, C. S., 2015	*	*	*	*	*		*	*	Medium
Kelly, T. N., 2015	*	*	*	*	**	*	*	*	High
Nicolas, A., 2015	*	*	*	*	*	*	*	*	High
Patente, T. A., 2015	*	*	*	*	*	*	*	*	High
Colares, V. S., 2014	*	*	*	*	*		*	*	Medium

Sandholm, N., 2013	*	*	*	*	*		*	*	Medium
Ozdemir, O., 2014	*	*	*	*	*		*	*	Medium
Sambo, F., 2014	*	*	*	*	*		*	*	Medium
Oguri, M., 2013	*	*	*	*	*		*	*	Medium
Ilic, V., 2014	*			*	*		*	*	Medium
Tavira, B., 2013	*	*	*	*	*		*	*	Medium
Hubacek, J. A., 2012	*		*	*	*		*	*	Medium
Karsli Ceppioglu, S., 2011	*			*	*		*	*	Medium
	*		*	*	*		*	*	Medium

Corredor, Z., 2020									
Lin, B. M., 2019	*	*	*	*	*		*	*	Medium
